# Cannabis Improves Obsessive-Compulsive Disorder—Case Report and Review of the Literature

**DOI:** 10.3389/fpsyt.2020.00681

**Published:** 2020-07-21

**Authors:** Natalia Szejko, Carolin Fremer, Kirsten R. Müller-Vahl

**Affiliations:** ^1^Clinic of Psychiatry, Social Psychiatry and Psychotherapy, Hannover Medical School, Hannover, Germany; ^2^Division of Neurocritical Care & Emergency Neurology, Department of Neurology, Yale School of Medicine, New Haven, CT, United States; ^3^Department of Bioethics, Medical University of Warsaw, Warsaw, Poland; ^4^Department of Neurology, Medical University of Warsaw, Warsaw, Poland

**Keywords:** obsessive-compulsive disorder, OCD, cannabinoids, cannabis-based medicine, endocannabinoid system, cannabis, depression

## Abstract

Although several lines of evidence support the hypothesis of a dysregulation of serotoninergic neurotransmission in the pathophysiology of obsessive-compulsive disorder (OCD), there is also evidence for an involvement of other pathways such as the GABAergic, glutamatergic, and dopaminergic systems. Only recently, data obtained from a small number of animal studies alternatively suggested an involvement of the endocannabinoid system in the pathophysiology of OCD reporting beneficial effects in OCD-like behavior after use of substances that stimulate the endocannabinoid system. In humans, until today, only two case reports are available reporting successful treatment with dronabinol (tetrahydrocannabinol, THC), an agonist at central cannabinoid CB1 receptors, in patients with otherwise treatment refractory OCD. In addition, data obtained from a small open uncontrolled trial using the THC analogue nabilone suggest that the combination of nabilone plus exposure-based psychotherapy is more effective than each treatment alone. These reports are in line with data from a limited number of case studies and small controlled trials in patients with Tourette syndrome (TS), a chronic motor and vocal tic disorder often associated with comorbid obsessive compulsive behavior (OCB), reporting not only an improvement of tics, but also of comorbid OCB after use of different kinds of cannabis-based medicines including THC, cannabis extracts, and flowers. Here we present the case of a 22-year-old male patient, who suffered from severe OCD since childhood and significantly improved after treatment with medicinal cannabis with markedly reduced OCD and depression resulting in a considerable improvement of quality of life. In addition, we give a review of current literature on the effects of cannabinoids in animal models and patients with OCD and suggest a cannabinoid hypothesis of OCD.

## Introduction

Obsessive-compulsive disorder (OCD) is characterized by the presence of obsessions and/or compulsions. Obsessions are defined as recurrent and persistent thoughts, urges, or images that are intrusive, unwanted, and cause—in most individuals—marked anxiety and/or distress. Compulsions are repetitive behaviors or mental acts that the person feels driven to perform in response to an obsession or according to the rules that must be applied rigidly (1). OCD is a severe and underdiagnosed mental disorder that causes substantial impairment in quality of life in the majority of patients. The prevalence of OCD is estimated to be around 2% to 3% (2–4). Up to this date, the only treatments approved for alleviation of OCD are cognitive-behavioral therapy (CBT) and pharmacotherapy with (selective) serotonin reuptake inhibitors ((S)SRI) (5). However, about one third of patients does not benefit from these treatment strategies, experiences recurrent episodes, or does not tolerate treatment with SSRI (6, 7). In these otherwise treatment-resistant patients surgical intervention using deep brain stimulation (DBS) has been suggested as an alternative option. Thus, novel treatments and new pharmacological components are urgently needed to improve outcome in patients with OCD.

The pathophysiology of OCD includes the involvement of different neurotransmitter systems, the most important being the serotoninergic system (8, 9). This serotonin hypothesis of OCD is mainly based on beneficial treatment effects of (S)SRI medication. In addition, a number of other pathways such as the GABAergic, glutamatergic, and dopaminergic have been suggested to be involved too (10–12). Accordingly, diverse brain regions have been suggested to be affected (13, 14). Only recently, the ENIGMA-OCD Consortium (15) reported less segregated organization of structural covariance networks in OCD, reorganization of brain hubs as well as a possible signature of altered brain morphometry especially in those regions involved in brain development and maturation such as the cingulate and orbitofrontal areas.

Against the background of increasing acceptance of cannabis-based medicines (CBM) in various diseases (16), increasing evidence for a paramount role of the central endocannabinoid system (ECS) in brain development (17) and stress regulation (18) as well as the fact that the ECS is the most important neuromodulatory system in the brain (19), research was initiated to explore the role of the ECS in the pathophysiology of OCD (20). Although only a small number of studies in animals and humans have been published addressing this topic, most of the data support the hypothesis that CBM might be effective in the treatment of OCD.

The aim of this report is to increase the database by presenting an illustrative case of a patient with severe OCD, who markedly benefitted from treatment with cannabis and, in addition, to summarize available scientific evidence supporting the importance of the ECS in the pathophysiology of OCD.

## Case Report

The male patient first presented in our outpatient clinic at the age of 22. Prenatal and perinatal development did not show any abnormalities and his medical history was unremarkable. Family history was negative without neurological and psychiatric diseases. Further history revealed that OCD symptoms already started in kindergarten age. At that time, he suffered from compulsions with the urge to constantly close the door and checking rituals accompanied by a just right feeling. During the following years, obsessions and compulsions exhibited a waxing and waning course without remission at any time. At the age of 17, in parallel to increased OCD symptoms, he developed a depressive episode with suicidal thoughts.

The diagnosis of OCD was made only one year before the first presentation in our clinic at the age of 21, after he had consulted his general practitioner, because his symptoms caused increasing problems in different areas of life, and he was worried that he would not be able to complete his training as animal keeper successfully. Therefore, pharmacotherapy with clomipramine was initiated with a maximal dose 25mg/d. Because of adverse events such as nausea and headache and because he felt no positive effect, he stopped medication after only one week.

During the first presentation in our clinic, the diagnosis of OCD according to DSM-5 was confirmed by one of the authors (KMV). At that time, the patient suffered from compulsions with repeated hand washing (up to 20×/day) and prolonged showering (up to 1h/day) including washing rituals as well as further rituals related to checking, ordering, and cleaning. In addition, he suffered from several obsessions such as contamination fear, sexual obsessions, repetition of same words, and magical thinking. He tried to neutralize these negative thoughts by imagining numbers. Several of these obsessions and compulsions were accompanied by a just right feeling. Altogether, he estimated to spent approximately 4 h a day on obsessive thoughts and compulsive behaviors. Non-surprisingly, OCD symptoms caused relevant problems in social life and at work. In his training as animal keeper, his boss and co-workers often exhorted him, mainly because of slowness due to his compulsions. At vocational school he had difficulties concentrating and learning. Due to his symptoms, he had stopped doing sports, neglected hobbies, avoided social contacts, and withdrew more and more socially. There was no evidence that his symptoms should be attributed to the effects of substance abuse or another medical condition.

The presentation in our clinic was motivated by the fact that he had noticed that use of cannabis alleviated his symptoms. He reported that he had started using cannabis recreationally at age 16. Because his symptoms markedly improved, he started to smoke street cannabis (mixed with tobacco) on a regular basis using 0.5 g cannabis three to four times per week (about 8 g/months). Use of street cannabis resulted in a constant and marked improvement of obsessions and compulsions of about 80% to 90% lasting for 12 to 15 h. In addition, his sleep improved because of a general feeling of relaxation, reduced OCD, and less rumination. No adverse events were reported. Despite these beneficial effects, he decided to stop using street cannabis on a regular basis at the age of 20, because he did not want to do anything illegal and because he was afraid of losing his driver’s license. Beside illegal use of street cannabis, the patient reported that he started smoking tobacco at the age of 15 (approximately 10 cigarettes/day). His further history of substance abuse was unremarkable. He reported not to drink any alcohol for several years.

We decided to initiate treatment with prescribed medicinal cannabis because of the diagnosis of severe OCD, due to the fact that the patient refused pharmacotherapy with (S)SRI as well as psychotherapy, and because of his reports of marked improvement after use of street cannabis. Before first prescription of medicinal cannabis, the patient stated that he has not smoked cannabis for about one year. Because THC content of street cannabis he had used before was unknown, we decided to test two different chemovars: *Bedrocan* containing 22% THC and <1% cannabidiol (CBD) and *Bedrobinol* containing 13.5% THC and <1% CBD. Both *Bedrocan* and *Bedrobinol* are produced by Bedrocan ^®^ Company in compliance with the European Medicines Agency’s good manufacturing practice (GMP) standards and ISO 9001: 2015 Certificates. Since the patient reported much better effects using *Bedrocan*, treatment with this chemovar was implemented with a daily dose of 0.2 to 0.3 g. Immediate thereafter, he reported a marked reduction of obsessions and compulsions of about 70% as well as general relaxation, improved sleep, and concentration at school as well as overall improvement of his quality of life resulting in better social functioning and reduced problems at work. For example, he restarted to practice sports. Twenty months after initiation of medicinal cannabis therapy, he passed his final theoretical and practical exams as animal keeper, and his employer offered him a permanent position. During the last months, he slightly increased the daily dose of *Bedrocan* cannabis up to 0.7 g.

At baseline, before implementation of treatment with medicinal cannabis as well as three, five, and 20 months later, a variety of clinical assessments have been performed by a clinical psychologist and psychotherapist (CF) experienced in this kind of tests. Detailed results are provided in **Table 1**. During the last follow-up visit, the patient reported an improvement of compulsions and obsessions of 90% to 95%. No side effects were reported. While in the past he had used street cannabis exclusively by smoking mixed with tobacco, after initiation of prescribed therapy with medicinal cannabis, he started inhale cannabis using a vaporizer in parallel. During the last follow-up visit, he reported to vaporize medicinal cannabis during the day and to smoke only once in the evening. However, for smoking he is mixing cannabis only with a small amount of tobacco (which is much smaller than in the past) and completely stopped smoking tobacco cigarettes.

**Table 1:** Clinical assessments at baseline and at different time points after treatment with medicinal cannabis.

Symptom	Scale (range)	Cutoff value	Baseline	Follow-up after baseline (% improvement compared to baseline)		
				3 months	5 months	20 months
OCD	Y-BOCS total (0–40) (21)	16	32	7 (78%)	10 (69%)	2 (94%)
	­ Y-BOCS obsessive subscore (0–20)	–	17	2 (88%)	4 (76%)	2 (88%)
	­ Y-BOCS compulsive subscore (0–20)	–	15	5 (67%)	6 (60%)	0
	OCI-R (0–72) (22)	18	40	10 (75%)	6 (85%)	2 (95%)
	OBQ-D (Descriptive scale) (23, 24)	severe, moderate, not clinically significant	severe	not clinically significant	not clinically significant	not clinically significant
Metacognitive beliefs	Metacognitions Questionnaire (30–120) (25)	–	137	77 (44%)	83 (39%)	74 (46%)
Depression	BDI (0–63) (26)	10	24	3 (88%)	1 (96%)	1 (96%)
Clinical global impression	CGI-S (0–7) (27)	–	5	3 (40%)	3 (40%)	2 (60%)
	CGI-I (0–7) (27)	–	–	2	2	1
QoL	QOLAS (0–50) (28)	–	25	8 (68%)	8 (68%)	6 (76%)
	SF-12*—Physical Functioning Subscale (0–100) (29)	–	41	55.26 (26%)	51.8 (29%)	55.3 (35%)
	SF-12*—Mental Health Subscale (0–100) (29)	–	23	57.89 (152%)	55.9 (143%)	57.9 (152%)

*the higher the score the better the quality of life.

OCD, obsessive compulsive disorder; OCI-R, obsessive-compulsive inventory—revised; OBQ-D, Obsessive-Beliefs Questionnaire—German version; QoL, quality of life; Y-BOCS, Yale-Brown Obsessive Compulsive Scale; BDI, Beck Depression Inventory; CGI-S, Clinical Global Impression—Severity Scale; CGI-I, Clinical Global Impression—Improvement Scale; QOLAS, The Quality of Life Assessment Schedule; SF-12, Short Form 12.

### Evidence Supporting the Role of the ECS in the Pathophysiology of OCD: Data Obtained From Animal Studies

Most robust data suggesting an involvement of the ECS in the pathophysiology of OCD comes from animal studies. Although the most widely used animal model for OCD is marble burying based on the observation that rats and mice will bury either harmful or harmless objects in their bedding, this model is not ideal. Critics point out that the marble burying test reacts to two types of drugs, (S)SRIs and benzodiazepines, although benzodiazepines have no effects in patients suffering from OCD. Therefore, findings from this animal model should be interpreted with caution. Rueda-Orozco et al. (30) showed that administration of the anandamide receptor antagonist AM251 delays extinction of OCD-like behavior in a procedural memory task in rats presumably caused by impaired endocannabinoid activity in the dorsolateral striatum. Gomes et al. (31) used a mouse model and demonstrated that activation of CB1 receptors using WIN55.212-2 results in reduced OCD-like behavior as indicated by a significant decrease in the number of buried marbles. They also applied an inhibitor of the anandamide hydrolysis that led to decreased marble burying. Nardo et al. (32) described attenuating influence of CBD on marble-burying behavior in mice. After administration of meta-chloro-phenyl-piperazine (mCPP), a substance that enhances OCD, animals were treated either with CBD (30 mg/kg) or the SSRI fluoxetine (10 mg/kg) resulting in similar reducing effects on marble burying. In contrast, Umathe et al. (33) reported increased marble-burying behavior in mice after use of high doses of the endocannabinoid anandamide and its analogues AM404 or URB597, while low doses of these substances decreased marble-burying. Deiana et al. (34) determined pharmacokinetic profiles of several phytocannabinoids after acute single-dose intraperitoneal and oral administration in mice and rats. The pharmacodynamic-pharmacokinetic relationship of CBD (120 mg/kg, intraparenchymal and oral) was further assessed using a marble burying test in mice. All phytocannabinoids penetrated similarly the blood-brain barrier. In rats, oral administration of CBD inhibited marble burying matching its pharmacokinetic profile. Casarotto et al. (35) also showed inhibitory effects of CBD on marble burying behavior in C57BL/6J mice. Varvel et al. (36) conducted an interesting experiment aiming to test the hypothesis that elevated brain levels of anandamide may potentiate extinction in a fixed platform water maze task. They used mice genetically deprived of the enzyme fatty acid amide hydrolase (FAAH) that inactivates anandamide. Accordingly, mice treated with the FAAH inhibitor OL-135 did not display any memory impairment or motor disruption but did exhibit a significant increase in the rate of extinction. FAAH compromised mice exhibited a significant increase in acquisition rate. The authors concluded, that endogenous anandamide facilitates extinction through a CB1 receptor mechanism of action and FAAH inhibition represents a promising pharmacological approach to treat disorders such as OCD. Further evidence from the field of genetics was published by Imperatore et al. (37), who used a monoacylglycerol lipase (MAGL) knock-out mouse as a genetic model of congenital and sustained elevation of 2-arachidonoylglycerol (2-AG) levels in the brain. MAGL(−) mice demonstrated impaired CB1 signaling and anxiety-like behavior. Finally, Kinsey et al. (38) showed that inhibition of the endocannabinoid catabolic enzymes FAAH and MAGL elicits anxiolytic-like effects in the marble burying assay.

### Studies in Patients With Pure OCD

Until today, only two case studies and one small controlled trial have been published reporting effects of CBM in a total of 14 patients with OCD (39–42). In 2008, Schindler et al. (41) described two patients with otherwise treatment-resistant OCD, who improved after adding dronabinol to preexisting treatments. The first patient was a 38-year-old woman with severe OCD and recurrent major depression, who had been treated with paroxetine (60 mg/d) and CBT with no improvement. Later on, therapy with clomipramine (300 mg/d) was initiated, which resulted only in a “partial response.” By chance, she discovered that smoking street cannabis improved her symptoms. Therefore, combined treatment with clomipramine and dronabinol (30 mg/per day) was started. After 10 days, OCD symptoms decreased by 50% (from 20 to 10 as measured by Yale-Brown Obsessive-Compulsive Scale, Y-BOCS). The second patient was a 36-year-old man with schizophrenia and OCD, who was admitted to hospital due to deterioration of psychotic and obsessive symptoms. The patient was previously treated with various antipsychotics such as haloperidol, olanzapine, risperidone, quetiapine, and aripiprazole as well as (S)SRIs without relevant effects. Even electroconvulsive therapy was performed without relevant improvement. After initiation of combined therapy with dronabinol (10 mg/d), clomipramine (150 mg/d) and clozapine (400 mg/d), OCD symptoms markedly improved within two weeks (from 23 to 15 according to Y-BOCS). Both patients reported no side effects.

In 2017, Cooper and Grant (40) reported about a 24-year-old male, who suffered a lacunar infarct of the left thalamus and thereafter developed persistent, repetitive, unwanted thoughts primarily related to doing unintentional harm or engaging in sexual acts with others. He had also a 10-year history of insulin-dependent diabetes and bipolar I disorder since age 16, which was stable on ziprasidone. His obsessions remained refractory to various agents used in monotherapy or combination (fluvoxamine, clomipramine, mirtazapine, risperidone, olanzapine, clozapine, ziprasidone, haloperidol, quetiapine, memantine, ondansetron, intravenous ketamine, *N*-acetylcysteine, gabapentin, clonazepam, plus mood stabilizing agents). Combination with dronabinol (20 mg/d) resulted in a marked improvement after two weeks of treatment (Y-BOCS score declined from 39 to 10) resulting in an amelioration of quality of life. In addition, for the first time he was able to tolerate CBT.

In 2020, the results of a trial were published using cannabinoid augmentation of exposure-based psychotherapy (EX) (39). Eleven unmedicated outpatients (18–60 years), who met DSM-5 criteria for OCD with at least moderate severity of symptoms (Y-BOCS ≥16), were assigned to either treatment with the synthetic THC analogue nabilone alone or a combination of nabilone+EX for 4 weeks. All participants received 1 mg nabilone twice a day orally. Nabilone was well tolerated, and no severe side effects occurred, but three participants withdrew because of increased anxiety. While monotherapy with nabilone resulted only in little symptom change (Y-BOCS decrease of 2.5 ± 3.6 after 4 months), combined treatment with nabilone+EX significantly improved the therapeutic effect of EX (Y-BOCS decrease of 11.2 ± 3.4) suggesting that nabilone can be used to augment treatment effects of EX in patients with OCD.

### Studies in Patients With Tourette Syndrome and Comorbid Obsessive-Compulsive Behavior

Further evidence supporting beneficial effects of CBM in OCD comes from clinical studies in patients with Tourette syndrome (TS), a complex spectrum disorder characterized by motor and vocal tics. Up to 80% of patients with TS, in addition, suffer from psychiatric comorbidities, most frequently obsessive-compulsive behavior (OCB) and attention deficit/hyperactivity disorder (ADHD). While there is increasing evidence from retrospective surveys, case studies, and small randomized controlled trials (RCTs) that different CBM improve tics in patients with TS, in some of these studies, an improvement of OCB has also been described (43–48).

In a case study in a 16-year-old male with TS and comorbid OCB, rage attacks, sleeping problems, anxiety, and depression treatment with dronabinol (up to 33.6 mg/d) resulted in an improvement of both tics and psychiatric comorbidities including OCB (37). In a survey among adult patients with TS (39), 17 of 64 patients reported use of marijuana. Of these, 14 reported a reduction of tics and one a remission of OCB after use of cannabis. In a retrospective analysis efficacy and safety of smoked cannabis was investigated in 19 adults with TS (36). Of these, 15 patients were also diagnosed with comorbid OCD. In all of them, OCD improved according to Y-BOCS after starting treatment with cannabis. Only recently, results of a retrospective analysis (n=98) and an online survey (n=40) have been published in patients with TS, who had used different kinds of CBM including street cannabis, the cannabis extract nabiximols, dronabinol, and medicinal cannabis (38). In patients with comorbid OCD, improvement of OCD symptoms of 15% to 42% was reported.

In a single-dose RCT (n=12) using 5.0, 7.5, or 10 mg THC, a significant improvement of OCB was found compared to placebo (mean reduction of 4.83 ± 5.59 according to the Tourette Syndrome Symptom List (TSSL), p=0.041) (47). In contrast, in an RCT using up to 10 mg THC/d over 6 weeks in 24 patients no improvement of OCD was found (48). A summary of all studies in animal and humans is shown in **Table 2**.

**Table 2:** Studies in animals and humans investigating the role of the endocannabinoid system (ESC) in OCD.

Reference	Research subjects (N, only in studies in humans)	Methods	Outcome
Rueda Orozco et al. (30)	Wistar albino male rats	Administration of the CB1 antagonist AM251 to enhance repetitive behavior Administration of anandamide	A dose–response blockade of extinction induced by AM251 No effect on repetitive behavior
Gomes et al. (31)	Male C57BL/6J mice	Activation of CB1 receptors using the CB1 agonist WIN55.212-2	Reduction of OCB
Nardo et al. (32)	Male swiss mice	Treatment with CBD compared to FLX following administration of mCPP, a substance that enhances OCD	Attenuating influence of CBD on MBB comparable with FLX
Umathe et al. (33)	Swiss mice	Administration of anandamide or its analogues AM404 and URB597	Increased MBB
Deiana et al. (34)	Wistar rats, Swiss mice	Determination of pharmacokinetic profiles of several phytocannabinoids after acute single-dose intraperitoneal and oral administration in mice and rats. Assessment of pharmacodynamic relationship after CBD administration and its influence on MBB test	All phytocannabinoids penetrated similarly the blood-brain barrier. In rats, oral administration of CBD inhibited OCB matching its pharmacokinetic profile.
Casarotto et al. (35)	C57BL/6J mice	Investigation of the effects of CBD, (S)SRI and DZ in C57BL/6J mice submitted to the MBB test	CBD, DZ, (S)SRI induced a significant decrease in the number of buried marbles compared with controls
Varvel et al. (36)	FAAH (−/−) mice, C57BL/6 mice	FAAH (+/+) and (−/−) mice were trained to acquire a fixed-platform water maze task Administration of THC Evaluation of THC effectiveness on extinction	The mice genetically deprived of the FAAH and mice treated with the FAAH inhibitor OL-135 did not display any memory impairment or motor disruption but did exhibit a significant increase in the rate of extinction. FAAH-compromised mice exhibited a significant increase in acquisition rate.
Imperatore et al. (37)	MAGL knock-out mouse	Creation of MAGL knock-out mouse (MAGL-/-), a genetic model of congenital and sustained elevation of 2-AG levels in the brain Evaluation of CB1R signaling	Genetic deletion of MAGL leaded to impaired cannabinoid receptor CB1R signaling and anxiety-like behavior.
Kinsey et al. (38)	C57BL/6J mice	Administration of DZ, THC, the MAGL inhibitor JZL184, the FAAH inhibitor PF-3845, the selective CB1 receptor antagonist rimonabant Testing of MBB under the influence of DZ, THC, JZL184, PF-3845	THC and DZ reduced MBB JZL184 and PF-3845 reduced MBB
Kayser et al. (39)	Human (11)	Nabilone augmentation of EX	Reduction of OCD symptoms
Cooper and Grant (40)	Human (1)	Treatment with dronabinol in otherwise treatment-resistant OCD	Improvement of OCD and quality of life
Schindler (41)	Human (2)	Treatment with dronabinol in otherwise treatment-resistant OCD	Improvement of OCD
M̈ller-Vahl et al. (46)	Human (17)	Retrospective analysis of clinical effects of smoked cannabis in patients with TS and comorbid OCB	N=1: remission of OCB
M̈ller-Vahl et al. (47)	Human (12)	RCT using single dose of THC	Significant improvement of OCB compared to placebo
M̈ller-Vahl et al. (48)	Human (24)	RCT using THC over 6 weeks	No improvement of OCD
Jakubovski and Muller-Vahl (44)	Human (2)	Effects of THC in TS with comorbid OCD	N = 1: Alleviation of tics, OCD and other comorbidities
Abi-Joude et al. (43)	Human (19)	Retrospective analysis investigating efficacy and safety of smoked cannabis in TS	In all of 15 patients with comorbid OCB at baseline, cannabis resulted in an improvement (according to Y-BOCS)
Milosev et al. (45)	Human (98 and 44)	Retrospective analysis and online survey in patients with TS who used CBM	Patients with comorbid OCD reported an improvement of OCD of 15–42%

OCD, obsessive-compulsive disorder; CBD, cannabidiol; FLX, fluoxetine; EX, exposure prevention therapy; TS, Tourette syndrome; RCT, randomized control trial; THC, tetrahydrocannabinol; CBM, cannabis based medicine; OCB, obsessive-compulsive behavior; Y-BOCS, Yale-Brown Obsessive Compulsive Scale; mCPP, meta-chloro-phenyl-piperazine; DZ, diazepam; MAGL, monoacylglycerol lipase; 2-AG, 2-arachidonoylglycerol; SSRI, serotonin selective reuptake inhibitor; FAAH, fatty acid amide hydrolase; MBB, marble burying behavior; N, number of participants.

## Discussion

The presented case report adds evidence to the hypothesis that modulation of the ECS by activating central CB1 receptors may improve OCD. Although in general our findings are in line with previous case reports (39–42), there are also some relevant differences: (i) while in all previous studies pure THC (dronabinol) or the synthetic analogue of THC, nabilone, have been used, our patient was treated with medicinal cannabis including more than 100 different cannabinoids; (ii) in previous case studies, preexisting treatment was augmented with CBM, while we used medicinal cannabis as monotherapy; and (iii) while all previously described patients were treatment resistant, in this case, the patient refused treatment with psychotherapy and had stopped medication with an (S)SRI due to adverse events after only one week. Thus, currently it is not only unclear whether CBM in general might be effective in the treatment of OCD, but also which cannabinoid or combinations of cannabinoids—and in particular the ratio of THC to CBD—is most effective and whether treatment with CBM should be used in monotherapy or in combination with (S)SRI or behavioral therapy.

Interestingly, neither our patient, nor those OCD patients described in the literature reported about clinically relevant adverse events. Most frequent adverse events associated with medically supervised treatment with THC and medicinal cannabis are dizziness, somnolence, drowsiness, problems with concentration, and tiredness, while severe adverse events occur only rarely. In general, CBM are well-tolerated, particularly when up-titrated slowly (49). It should be noted that our patient had used street cannabis (without negative effects) for several years, before treatment with medicinal cannabis was initiated. Thus, it cannot be excluded that CBM might be effective only in a subgroup of patients with OCD, who tolerate this kind of treatment well. In any case, before initiating treatment with CBM contraindications such as psychosis must be excluded, and patients must be informed about potential adverse events.

Currently, possible underling mechanisms of beneficial effects of CBM in OCD are unknown. However, it can be speculated that positive effects might be related to the well-known complex interplay between cannabinoids and serotonin (5-HT) function. In *in vivo* and *in vitro* studies, it has been demonstrated that cannabinoids modulate the activity of the 5-HT system at several levels. For example, it has been shown that activation of CB1 receptors affects the synthesis and release of 5-HT and that CB1 receptor agonists—depending on dose and duration of administration—either reduce or increase firing activity in 5-HT cells in the dorsal raphe nucleus [for review see: (50)]. Thus, based on the serotonin hypothesis of OCD, it can be speculated that CBM improves OCD by modulating the serotoninergic system. However, one might also speculate that OCD is caused by a dysfunction within the ECS. Accordingly, CBM might ameliorate OCD *via* direct activation of the ECS or indirectly by reducing anxiety (51) and stress (52). In line with this hypothesis, it has been demonstrated that chronic stress leads to a downregulation of CB1 receptor signaling in brain regions involved in OCD such as hippocampus, striatum, nucleus accumbens, prefrontal cortex, dorsal raphe nucleus, amygdala, and hypothalamus [for review see (18)]. Furthermore, an endogenous molecular mechanism has been identified in a specific cortico-striatal pathway that mediates the transition between goal-directed and habitual action strategies (53). Deletion of CB1 receptors from orbital frontal cortex neurons projecting to the dorsal striatum prevents mice from shifting from goal-directed to habitual action control suggesting that the emergence of habits depends on endocannabinoid-mediated attenuation.

However, we cannot entirely exclude that beneficial effects in the presented case are only caused by placebo effects and positive expectations or other possible confounders such as environmental, emotional or psychological factors that might have influenced OCD. It also cannot be ruled out that treatment with medicinal cannabis only indirectly influenced OCD symptoms by reducing stress or improving other symptoms such as anxiety, depression or sleeping problems.

## Conclusion

There is increasing evidence that the ECS might be involved in the pathophysiology of OCD. In line with this hypothesis, from a limited number of case studies it is suggested that CBM might be effective in the treatment of OCD. However, so far it is unclear, which cannabinoids—in monotherapy of combination with other treatments—might be most effective and which patients might respond best.

## Data Availability Statement

All datasets presented in this study are included in the article/supplementary material.

## Ethics Statement

Ethical review and approval was not required for the study on human participants in accordance with the local legislation and institutional requirements. The patients/participants provided their written informed consent to participate in this study. Written informed consent was obtained from the individual for the publication of any potentially identifiable images or data included in this article.

## Author Contributions

KM-V and CF conceived and designed the study and acquired data. CF set up the electronic database. KM-V, NS and CF interpreted the data and reviewed and edited the manuscript. KM-V and NS wrote the original draft of the manuscript.

## Conflict of Interest

The authors declare that the research was conducted in the absence of any commercial or financial relationships that could be construed as a potential conflict of interest.
